# Alpha-Linolenic Acid-Enriched Butter Promotes Fatty Acid Remodeling and Thermogenic Activation in the Brown Adipose Tissue

**DOI:** 10.3390/nu12010136

**Published:** 2020-01-03

**Authors:** Mikyoung You, Rong Fan, Judy Kim, Seung-Ho Shin, Soonkyu Chung

**Affiliations:** 1 Department of Nutrition and Health Sciences, University of Nebraska-Lincoln, Lincoln, NE 68588, USA; myou3@unl.edu (M.Y.); rfan4@unl.edu (R.F.);; 2 Sunseo Omega 3 Inc., University of Nebraska Innovation Campus, Lincoln, NE 68588, USA; chairman@somega3.com

**Keywords:** alpha-linolenic acid, bio-fortification, butter, brown thermogenesis, brown fat, elongase 6

## Abstract

Supplementation with *n*-3 long-chain (LC) polyunsaturated fatty acids (PUFA) is known to promote thermogenesis via the activation of brown adipose tissue (BAT). Agricultural products that are biofortified with *α*-linolenic acid (ALA), the precursor of *n*-3 LC PUFA, have been launched to the market, but their impact on BAT function is unknown. This study aimed to evaluate the effects of ALA-biofortified butter on lipid metabolism and thermogenic functions in the BAT. C57BL/6 mice were fed a high-fat diet containing ALA-biofortified butter (n3Bu, 45% calorie from fat) for ten weeks in comparison with the isocaloric high-fat diets prepared from conventional butter or margarine. The intake of n3Bu significantly reduced the whitening of BAT and increased the thermogenesis in response to acute-cold treatment. Also, n3Bu supplementation is linked with the remodeling of BAT by promoting bioconversion into *n*-3 LC PUFA, FA elongation and desaturation, and mitochondrial biogenesis. Taken together, our results support that ALA-biofortified butter is a novel source of *n*-3 PUFA, which potentiates the BAT thermogenic function.

## 1. Introduction

Adult humans contain a significant amount of metabolically active brown adipose tissue (BAT), which is estimated to be responsible for ~20% of total energy expenditure through non-shivering thermogenesis (adaptive thermogenesis) [1,2,3]. The loss of BAT mass or activity is positively correlated with the prevalence of obesity and type 2 diabetes [4,5]. Conversely, increasing thermogenic activity, either by the transplantation of metabolically active BAT or by pharmacological treatment with beta-3 adrenoceptor agonists, alleviates the symptoms of type 2 diabetes [6,7]. Also, accumulating evidence suggests that the maintenance of BAT function is a viable strategy to circumvent obesity-mediated metabolic dysfunction [8,9].

Several bioactive compounds derived from our foods have shown to modulate brown thermogenesis [10,11]. Dietary fatty acids are not only a primary fuel for energy production but also critical regulators of BAT function. Previously, our group demonstrated that the consumption of dietary saturated fatty acids causes Toll-like receptor 4 (TLR4)-mediated ER stress and mitochondrial dysfunction, leading to an attenuation of adaptive thermogenesis [12]. In contrast, the intake of fish oil, a significant source of *n*-3 long-chain (LC) polyunsaturated fatty acids (PUFA), such as eicosapentaenoic acid (EPA) and docosahexaenoic acid (DHA), potentiates the cell-autonomous brown adipocyte differentiation from murine brown precursor cells [13,14]. Additionally, maternal fish oil supplementation during pregnancy promotes prenatal BAT formation in vivo, which may decrease the risk of childhood obesity by augmenting BAT-mediated energy expenditure [15]. Moreover, we have demonstrated that fish oil consumption facilitates the thermogenic potential of BAT, resulting in improved insulin sensitivity against diet-induced obesity and insulin resistance [12,13].

Despite the various metabolic benefits, including thermogenic energy expenditure, fish oil has limited application as a routinely consumed food, due to unpleasant fish odor. Hence, there is a growing demand for a new source of *n*-3 PUFA replacing fish products. Numerous attempts have been made to generate the *n*-3 PUFA-containing agricultural products, either through the incorporation of fish oil into dairy products via enzymatic inter-esterification [16,17,18] or through the bio-fortification of farm animals by providing *n*-3 PUFA-rich feed [19]. Instead of fish oil, plant-based *n*-3 PUFA (i.e., ALA) is frequently used for feedstuff, as plant-based *n*-3 PUFA is absent of fish odor and inexpensive compared to fish-based *n*-3 LC PUFA (i.e., DHA or EPA). Dairy products produced by bio-fortified cows have been launched to the market, such as *n*-3 PUFA-fortified milk and butter. However, its efficacy on brown thermogenesis is largely unknown.

Recently, we reported that ALA-enriched butter is effective in attenuating HF diet-induced insulin resistance and hepatic steatosis by promoting the endogenous bioconversion of ALA into *n*-3 LC PUFA [20]. In that study, we focused on evaluating the impact of ALA-enriched butter on lipid metabolism and thermogenic function of BAT by using the same C57BL/6 mice that we reported previously [20]. Here, we thoroughly assessed the metabolic changes in the BAT upon chronic consumption of ALA-enriched butter in comparison with the isocaloric HF feedings prepared from conventional butter or margarine.

## 2. Materials and Methods

### 2.1. Animals and Diet Preparation

All animals were maintained in accordance with the protocols approved by the Institutional Animal Care and Use Committee (IACUC) at the University of Nebraska–Lincoln. C57BL/6 mice were purchased at 6 weeks of age from Jackson Laboratory. The mice were randomly assigned into four groups (*n* = 8/group)—chow group (chow) or isocaloric high-fat diet groups (45% total calories from fat) from conventional butter (Bu, Highland Dairy Foods), *n*-3 enriched butter (n3Bu, Sunseo Omega Inc.), or margarine (Ma, Land O’Lakes)—and fed for 10 weeks *ad libitum*. The diets were formulated based on the AIN-93M purified rodent formula (diet composition in Table S1). The bodyweight and food intake were measured weekly.

### 2.2. Acute Cold Treatment and Rectal Temperature

To measure the thermogenic potential, the mice were exposed to cold temperatures (4 °C) acutely (3 h). The core body temperature was detected using a rectal probe for mice (RET-3 rectal probe for mice, Kent Scientific Corporation, Torrington, CT, USA). An infrared (IR) camera (A655sc, FLIR Systems Inc., Wilsonville, OR, USA) was used to detect thermal release and capture images of the surface body temperature. FLIR Research IR program software was used to display surface heat release via a color palette representing temperatures between 10 and 34 °C. To determine the fatty acid profile change during cold stress, the mice were housed either at room temperature (Rm, 22 °C) or a cold temperature (Cold, 4 °C) for 48 h. At the time of necropsy, brown adipose tissue (BAT) was collected, snap-frozen in liquid nitrogen, and kept at −80 °C until analysis.

### 2.3. Fatty Acid Profile of Diet and BAT

To determine the FA profile in the BAT, the total lipids were extracted, as we previously described [15]. Briefly, approximately ~100 mg of BAT was minced, and the extracted total lipids were converted into fatty acid methyl ester. Gas chromatography was performed on Agilent Technologies using a capillary HP-88 column (100 m × 0.25 mm × 0.2 μm film thickness). The identity of the lipid species was determined by comparing its relative retention times with the commercial mixed-FA standard (NU-CHEK PREP). The area percentages for all resolved peaks were analyzed using the ChemStation Software (Agilent Technologies, Santa Clara, CA, USA). To calculate the C18:C16 and SCD ratio, we used the formulation below.

Elongation (C18:C16) ratio = (C18:0 + C18:1n7 + C18:1n9)/(C16:0 + C16:1n7)(1)

SCD ratio = (C18:1n7 + C18:1n9 + C16:1n7)/(C16:0 + C18:0)(2)

### 2.4. qPCR and Quantification of mtDNA/gDNA Ratio

The total RNA was extracted from ~50 mg of BAT using TRIzol™ Reagent and treated with the DNA-free™ DNA removal kit (ThermoFisher Scientific, Waltham, MA, USA). Then, the RNA was reverse transcribed for cDNA synthesis (iScript, BioRad, Hercules, CA, USA). Real-time PCR was carried out on a QuantStudio 6 Flex (Applied Biosystems, Foster City, CA, USA) using SYBR Green (Fisher scientific). Equal amounts of cDNA prepared from each individual animal were pooled (*n* = 4 per group) and qPCR reactions were performed in triplicate. The relative gene expression was calculated based on the 2^−ΔΔCT^ method with the normalization of the raw *Ct* values to *Hprt* (hypoxanthine-guanine phosphoribosyltransferase) (Figures S1 and S2). The primer sequences are available in Table S2. To determine the mitochondrial DNA to genomic DNA ratio, the total DNA was isolated using DNAzol (Life Technologies, Gaithersburg, MD, USA), as we described previously [21].

### 2.5. Western Blot Analysis

The protein was extracted from BAT using a RIPA buffer containing protease and phosphatase inhibitors (Sigma, St. Louis, MO, USA). Proteins were fractionated using 10% SDS-PAGE, transferred to PVDF membranes, and incubated with antibodies agonist uncoupling protein 1 (UCP1), PR-domain containing 16 (PRDM16), CD11c, F4/80, stearoyl-Coenzyme A desaturase 1 (SCD-1), elongation of long-chain fatty acid-like family member 6 (Elovl6), voltage-dependent anion channel 1 (VDAC1), pyruvate dehydrogenase (PDH), respiratory oxidative phosphorylation protein (OxPhos), and *β*-actin. Chemiluminescence from ECL solution (Western Lightning) was detected using an ODYSSEY FC Imaging System (LI-COR). The details of antibody information are available in Table S3.

### 2.6. H&E Staining

The BAT samples were fixed in 10% buffered formalin, embedded in paraffin, and cut into 5 μm sections. After deparaffinization, BAT sections were stained with hematoxylin and eosin (H&E) staining as previously described [15].

### 2.7. Statistics

All data were analyzed using one-way ANOVA followed by Tukey′s multiple comparison tests or Student′s *t*-test, * *p* < 0.05 and *** *p* < 0.001. All analyses were performed using Graph Pad Prism (Version 6.02).

## 3. Results

### 3.1. Supplementation with ALA-Biofortified Butter Promoted Thermogenic Potential in the BAT

**Figure 1:**
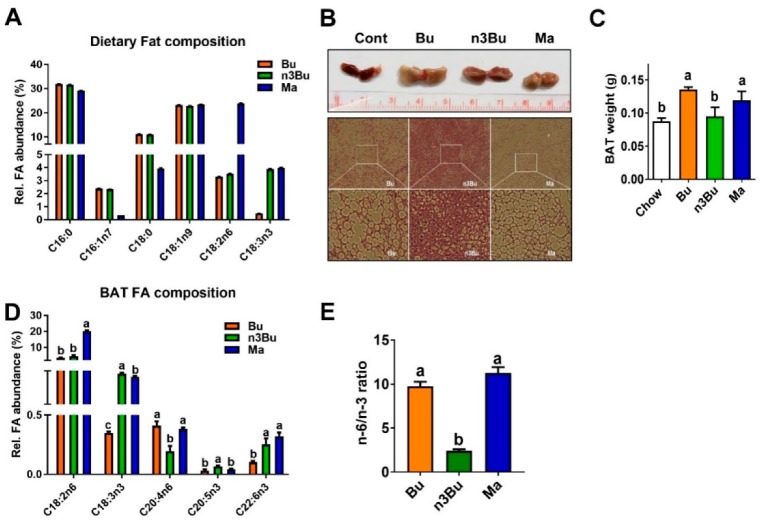
Supplementation with *α*-linolenic acid (ALA)-biofortified butter decreased triglyceride accumulation and *n*-6/*n*-3 polyunsaturated fatty acids (PUFA) ratio in the brown adipose tissue (BAT). (**A**) Fatty acid composition of the isocaloric high-fat (HF) diets made of conventional butter (Bu), ALA-biofortified butter (n3Bu), and margarine (Ma). (**B**) Gross image (upper) and histology of BAT (lower) after 10 weeks of HF diet feeding (representative of *n* = 8/group). (**C**) BAT weight. (**D**) Fatty acid profile in the BAT after supplementation. (**E**) *n*-6/*n*-3 PUFA ratio in the BAT. All data represented as mean ± SEM. Treatments with different letters are significantly different from one another (*p* < 0.05) by one-way ANOVA with Tukey’s multiple comparison tests.

Fatty acid (FA) analysis by GC/MS revealed that butter made out of biofortified milk (refer to as bio-fortified butter) contained approximately ~4% of ALA (C18:3), similar to margarine, while conventional butter was nearly absent of ALA. Except for ALA content, FA composition was identical between conventional butter and *n*-3 PUFA fortified butter. Made out of vegetable oil (~80%), margarine contained a lower amount of saturated FA (both C16:0 and C18:0) and palmitoleic acid (C16:1n7), but possessed a 10-fold higher amount of linoleic acid (LA, C18:2) than conventional butter or ALA-biofortified butter. In all samples, other PUFA levels, such as arachidonic acid (20:4, ARA), DHA, and EPA, were negligible (Figure 1A).

The C57BL/6 mice were fed for ten weeks with one of the isocaloric high-fat diets prepared from conventional butter (Bu), ALA-biofortified butter (n3Bu), and margarine (Ma). HF feeding with Bu or Ma, but not n3Bu, significantly increased the BAT weight compared to chow (Figure 1B,C). H&E staining of BAT section revealed that feeding with Bu or Ma remarkably induced white adipocyte-like morphological changes in the BAT, but a significantly lesser degree was found with n3Bu feeding (Figure 1B). Reflecting the dietary LA content, the Ma diet induced a ~2-fold increase in LA levels in the BAT. Intriguingly, 10 weeks of the n3Bu diet significantly reduced ARA content, while promoting the EPA content in the BAT compared with the Bu or Ma diet (Figure 1D). Consequently, n3Bu feeding decreased the intracellular *n*-6/*n*-3 FA ratio in the BAT by ~4-fold compared to Bu or Ma (Figure 1E). These results suggest that the bioconversion of ALA to *n*-3 LC PUFA is facilitated in n3Bu-fed BAT compared to Ma-fed BAT.

**Figure 2:**
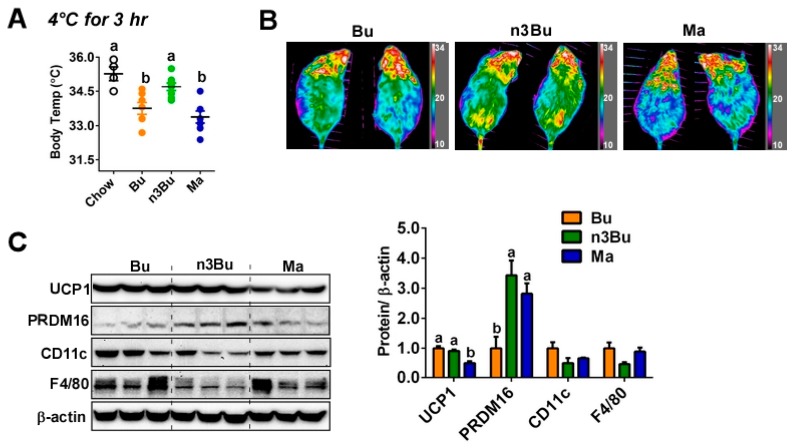
Supplementation with ALA-biofortified butter promoted thermogenesis and suppressed inflammation in the BAT. (**A**) Core body temperature after exposing animals to 4 °C acutely for 3 h (*n* = 4 for chow, *n* = 8 for HF-fed animals). (**B**) Heat release captured by IR camera at the end of a 3-h cold exposure. (**C**) Western blot analysis of UCP1, PRDM16, CD11c, and F4/80. *β*-actin used as a loading control for quantification. All data represented as mean ± SEM. Treatments with different letters are significantly different from one another (*p* < 0.05) by one-way ANOVA with Tukey’s multiple comparison tests.

Next, we asked whether the dietary reduction of the *n*-6/*n*-3 FA ratio in ALA-biofortified butter alters BAT thermogenic activity. When mice were placed to a cold temperature (4 °C) acutely (3 h), n3Bu-fed mice were able to maintain a higher core-body temperature than Bu- or Ma-fed mice, as comparable to chow-fed mice (Figure 2A). The heat release, captured by IR camera, was higher in n3Bu-fed mice than Bu- or Ma-fed mice (Figure 2B). The qPCR analysis of brown-specific gene expressions revealed that Ma feeding substantially decreased the brown signature gene expressions of *Ucp1*, *Prdm16*, *Pgc1α*, *Cidea*, and *Dio2*, while n3Bu showed a tendency to increase these gene expressions compared to the Bu control (Figure S1). Consistently, the protein expression levels of uncoupling protein 1 (UCP1) were reduced in Ma-fed mice compared to Bu or n3Bu. The expression levels of PRDM16, a key transcription factor for brown adipogenesis, were higher in n3Bu-fed BAT than Bu- or Ma-fed BAT. Importantly, BAT from n3Bu feeding showed a remarkable decrease in F4/80 and CD11c expression levels, implying that the n3Bu suppresses macrophage infiltration and proinflammatory M1 polarization compared to the Bu- or Ma-fed group (Figure 2C).

### 3.2. Supplementation with ALA-Biofortified Butter Altered FA Composition in the BAT

**Figure 3:**
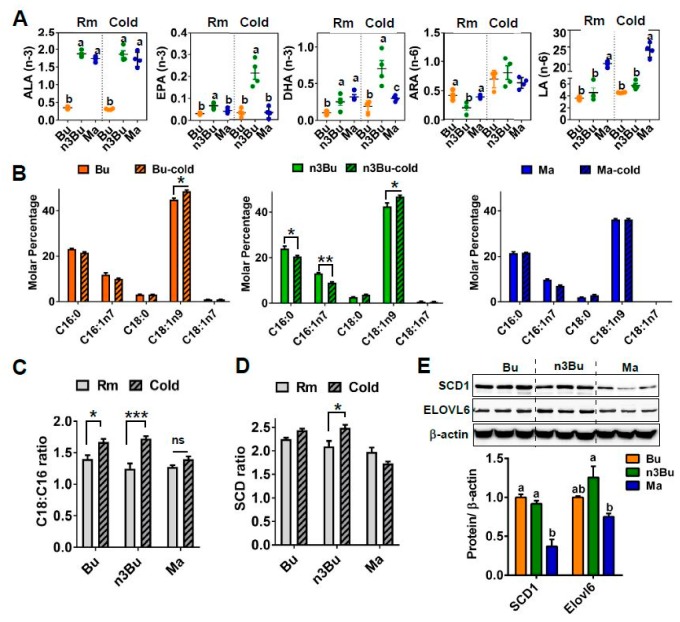
Supplementation with ALA-biofortified butter increased *n*-3 PUFA content and degree of desaturation and elongation of fatty acids in the BAT in response to cold exposure. (**A**) Composition of *n*-3 (ALA, EPA, and DHA) and *n*-6 (ALA and LA) PUFA in the BAT at room temperature (Rm) or cold exposure (Cold) for 48 h (*n* = 4 per group). (**B**) Fatty acid methyl ester analysis of BAT at Rm and Cold. (**C**) C18:C16 ratio. (**D**) SCD ratio. (**E**) Western blot analysis of stearoyl-CoA desaturase 1 (SCD-1), elongation of long chain fatty acid-like family member 6 (ELOVL6). Each lane represents an individual animal (*n* = 3) and the *β*-actin used as a control for quantification (below). In (**A**,**E**), treatments with different letters are significantly different from one another (*p* < 0.05) by one-way ANOVA. All data represented as mean ± SEM. In (**B**–**D**), * *p* < 0.05, and *** *p* < 0.001 by Student′s *t*-test.

Emerging evidence suggests that the thermogenic activation of BAT is associated with FA remodeling, including augmented *n*-3 LC PUFA synthesis and FA elongation [22]. Based on this literature suggestion, we next investigated the impact of n3Bu on lipid metabolism in the BAT during thermogenic activation. The GC/MS analysis revealed that cold treatment (CT, 4 °C) for 48 h resulted in a rapid increase in EPA and DHA in the BAT in response to n3Bu feeding compared to mice kept at an ambient temperature (RT, 22 °C) (Figure 3A). However, these changes in *n*-3 LC PUFA were less prominent in the BAT with Bu feeding and almost completely absent with Ma feeding (Figure 3A). We also examined the differential impact of Bu, n3Bu, and Ma feeding on FA elongation and desaturation. Cold treatment decreased the content of C16 FA (palmitic and palmitoleic acid) but increased the C18 FA (stearic acid) content in n3Bu-fed BAT and, to a lesser degree, Bu-fed BAT. However, Ma-fed BAT failed to alter the FA elongation in response to cold temperatures (Figure 3B). Therefore, the FA elongation ratio (see the formula in the method section) in the BAT was most evident with n3Bu feeding (Figure 3C). Also, the degree of FA desaturation was higher in n3Bu-fed BAT than in Bu- or Ma-fed BAT upon cold treatment (Figure 3D). The reductions in SCD-1 and ELOVL6 expression in Ma-fed BAT were significant (Figure 3E), although the difference between n3Bu- and Bu-fed BAT did not reach statistical significance. Consistently, there was an increase in the transcription levels of *Scd-1*, *Elovl6*, and *Elvol3* in the n3Bu-fed BAT compared to Bu, but a substantial decrease in these genes in Ma-fed BAT (Figure S2). However, the changes in the delta-5 and delta-6 desaturase levels were similar between the groups (Figure S2). Collectively, these results suggest that ALA-biofortified butter facilitates the cold-mediated *n*-3 LC PUFA synthesis and FA elongation/desaturation.

### 3.3. Supplementation with ALA-Biofortified Butter Facilitated Mitochondrial Biogenesis

**Figure 4:**
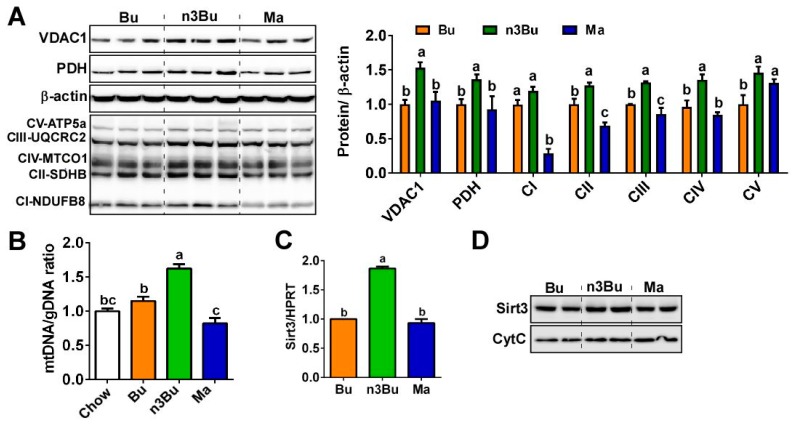
Supplementation with ALA-biofortified butter increased mitochondrial biogenesis in the BAT. (**A**) Western blot analysis of mitochondrial proteins of voltage-dependent anion channel 1 (VDAC1), pyruvate dehydrogenase (PDH), and respiratory protein complexes I-V (left). *β*-actin was used as a control for quantification (right). (**B**) mtDNA to gDNA ratio in BAT by qPCR (*n* = 6 per group). (**C**) *Sirt3* mRNA expression in BAT by qPCR (*n* = 6 per group). (**D**) Western blot analysis of Sirt3 in the mitochondrial fraction. Each lane represents individual animals in duplication. Cyt C was used as a control. All data represented as mean ± SEM. Treatments with different letters are significantly different from one another by one-way ANOVA (*p* < 0.05).

Molecular events for thermogenic activation include mitochondrial biogenesis in the BAT [23]. Next, we investigated whether the improved thermogenic function by n3Bu feeding is linked with mitochondrial biogenesis. There was a substantial increase in mitochondrial proteins in the n3Bu-fed BAT compared to Bu- or Ma-fed BAT, including (1) voltage-dependent anion channel 1 (VDAC1) located in the mitochondrial outer membrane, (2) pyruvate dehydrogenase (PDH) located in the mitochondrial matrix, and (3) oxidative phosphorylation proteins (OxPhos) located in the mitochondrial inner membranes (Figure 4A). Conforming to the increased mitochondrial mass, the mitochondrial DNA to genomic DNA ratio (mtDNA/gDNA) was significantly higher in n3Bu-fed BAT compared to Bu- or Ma-fed BAT (Figure 4B).

Emerging evidence also suggests that sirtuin 3 (SIRT3), a NAD^+^-dependent deacetylase in mitochondria, is a key modulator for brown thermogenesis [24]. Consistent with this study, the transcriptional levels of *Sirt3* and SIRT3 protein expression levels were higher in n3Bu-fed BAT than Bu or Ma-fed BAT (Figure 4C,D). Collectively, these data support the assertion that ALA-biofortified butter effectively promotes thermogenesis, partly through the facilitation of mitochondrial biogenesis in the BAT.

## 4. Discussion

**Figure 5:**
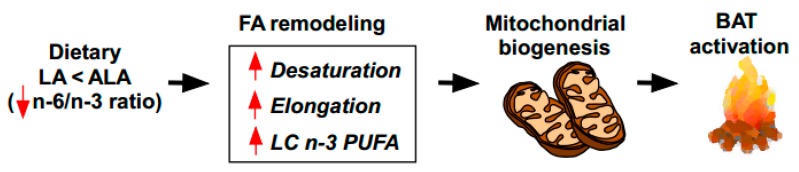
Working model of dietary ALA enrichment on thermogenic activation in the BAT. The reduced *n*-6/*n*-3 ratio by dietary supplementation with ALA enriched foods induces fatty acid remodeling, including the augmentation of desaturation, elongation, and long-chain (LC) *n*-3 PUFA formation. These FA profile changes facilitate mitochondrial biogenesis, leading to the thermogenic activation of the BAT.

Previously, we have reported that ALA-enriched butter was effective in attenuating HF diet-induced insulin resistance compared to the other isocaloric diets prepared from conventional butter or margarine [20]. The metabolic improvement by ALA-biofortified butter was associated with augmented bioconversion into *n*-3 LC PUFA, reduced inflammation in the metabolic tissues (i.e., liver and WAT), and systemic production of anti-inflammatory oxylipins [20]. Here we investigated the impact of ALA-biofortified butter in regulating the BAT thermogenesis. The present work demonstrated that ALA-biofortified butter attenuated HF diet-mediated BAT whitening and inflammation (Figure 1), and increased the brown fat specific gene and protein markers and thermogenic activity (Figure 2). In terms of mechanism, the intake of ALA-biofortified butter facilitates cold-mediated lipid remodeling by promoting *n*-3 LC PUFA conversion, by increasing ELOVL6 and SCD activity and by stimulating mitochondrial biogenesis (proposed working model in Figure 5). Taken together, our work suggests that ALA-biofortified butter could be an alternative to fish oil in activating brown thermogenesis.

Accumulating evidence suggests that FA are critical modulators of non-shivering adaptive thermogenesis [25]. At least three different modes of FA regulation have been reported to promote thermogenic regulation in the BAT, including (1) increased *n*-3 LC PUFA levels [13,26], (2) increased FA elongation and desaturation [22], and (3) increased cardiolipin (CL) synthesis [27,28]. Here, we discuss our results based on these FA regulations for thermogenic activation.

The endogenous synthesis of *n*-3 LC PUFA requires a series of reactions by elongases and desaturases. In general, LC *n*-6 PUFA synthesis is the favored pathway over *n*-3 PUFA formation due to the overabundance of LA to ALA [29,30]. It is important to note that BAT possesses a unique feature of increasing the *n*-3 LC PUFA content in response to thermogenic stimuli [26]. Our previous results showed that fish oil supplementation elevated the *n*-3 LC PUFA content upon cold treatment [13]. Similarly, the supplementation with ALA-biofortified butter induced an increase in *n*-3 LC PUFA levels in basal as well as cold-stimulated conditions (Figure 3A). The increased bioconversion from ALA to *n*-3 LC PUFA (i.e., DHA and EPA) is dependent on the reduced dietary *n*-6/*n*-3 PUFA ratio. Despite having the same ALA content, margarine supplementation was unable to increase *n*-3 LC PUFA content, presumably due to high LA content (Figure 3A). The precise mechanism by which cold treatment promotes bioconversion of *n*-3 LC PUFA in the BAT is yet to be determined. One mechanism could be the induction of GRP120, a well-known membrane receptor for *n*-3 PUFA, in the BAT during cold exposure [31]. It is possible that *n*-3 PUFA released from the WAT are redistributed into the BAT through GRP120. This scenario could increase the *n*-3 PUFA levels in the BAT independent of transcriptional modulation of delta-5 or delta-6 desaturase, the crucial enzymes for PUFA synthesis. Our system may fit in this scenario, as ALA-biofortified butter consumption promoted cold-induced *n*-3 PUFA content without modification of the mRNA levels of *Fads1* and *Fads2* (Figure S2). Nonetheless, our results strongly support the assertion that increased availability of *n*-3 PUFA in the BAT upon intake of ALA-biofortified butter stimulates thermogenic activation.

In addition to an increase in *n*-3 LC PFUA content, BAT should undergo other compositional changes in FA in order to activate BAT, including the modulation of FA elongation and desaturation [26]. The enzyme ELOVL6 has been demonstrated to regulate thermogenic capacity; Tan et al. revealed that cold treatment significantly promotes the C18:C16 ratio in the BAT due to ELOVL6 activity [22]. In parallel, thermogenic activation of BAT is associated with an elevated desaturase index and SCD-1 activity. Conversely, genetic ablation of *Elovl6* was unable to induce a full thermogenic recruitment of BAT. More interestingly, the activation of ELOVL6 activity in the BAT is linked with mitochondrial function [32], suggesting that ELOVL6 activity is required for the remodeling of mitochondria for enhancing thermogenic potential. Consistent with this support from the literature, our results show that the indices of cold-induced FA elongation and desaturation were higher with ALA-biofortified butter than conventional butter or margarine (Figure 3C). These FA compositional changes by ALA-biofortified butter consumption were correlated with enhanced mitochondrial biogenesis (Figure 4), implying that FA remodeling is required for mitochondria in the BAT. Recently, Sebaa et al. demonstrated that an increase in mitochondrial deactylation by SIRT3 plays a key role in UCP1 regulation for thermogenic activation. ALA-biofortified butter increased the SIRT3 protein and gene expression in the mitochondria fraction in BAT, suggesting that ALA-biofortified butter may upregulate the deacetylation. We are currently investigating the mechanism by which increased *n*-3 PUFA levels promote SIRT3 activation and mitochondrial deacetylation.

Cardiolipins (CL) are unique phospholipids that are predominantly found in the mitochondrial inner membrane. CL have critical functions in the formation of the respiratory supercomplex [33,34]. Lipidomic analysis revealed that cold adaptation induces de novo CL synthesis as well as the remodeling of CL with the longer and less saturated acyl chains in the BAT [28]. These results are consistent with our observation that ALA-biofortified butter increased ELOVL6 and SCD-1 activity in the BAT. Unfortunately, we were unable to measure the CL lipid content due to a shortage of BAT samples. Also, no significant differences were found in mRNA expression levels of CL synthase (*Crls1*) among the isocaloric dietary groups (Figure S3). Nonetheless, we speculate that the CL species would be different in ALA-biofortified butter-fed BAT based on an enhanced FA elongation and desaturation. It is of interest to determine the impact of differential dietary fat intakes on mitochondrial CL species regulation and its association with mitochondrial thermogenic capacity as a future research.

In addition, there is growing evidence showing that conjugated linoleic acid (CLA), either trans-10 or cis-12 isomer alone, or the mixture with cis-9 and trans-11 isomer, increases the thermogenic activity in the adipose tissue [35,36,37]. The thermogenic action of CLA is most evident in the WAT with a minor impact on the BAT [37]. It is quite distinctive from the thermogenic activity exerted by ALA enrichment in this work, since n3Bu supplementation promotes thermogenesis in the BAT, while causes a minimal impact on the WAT. It would be of interest to investigate whether CLA supplementation also induces similar FA remodeling such as augmented desaturation, elongation and bioconversion of LC *n*-3 PUFA.

Bio-fortification of *n*-3 PUFA is widely provided to farm animals such as pigs, chicken, and cows by supplementing the feed with a plant source of *n*-3 PUFA, i.e., flaxseed oil or algae [38]. However, the endogenous conversion rate from ALA to *n*-3 PUFA is very low in ruminant animals. The most significant barrier is suppressing the bio-hydrogenation of ruminal microbes, which revert dietary unsaturated FA into SFA in the rumens. Without the modulation of gut microbes, the dietary unsaturated FA are unable to reach the small intestine for absorption and incorporation [39]. Numerous efforts have been made to increase *n*-3 PUFA content in cattle in the hope of yielding *n*-3 PUFA-enriched dairy products such as milk, cheese, and butter [40]. For example, the generation of transgenic cattle was attempted by introducing the *Fat-1* gene, the *n*-3 FA desaturase derived from *C. elegans* [41]. However, this approach is unfavorable to customers based on an unavoidable dispute regarding the health concerns related to genetically-modified foods. Industrial incorporation of fish oil into dairy products by enzymatic inter-esterification also has limitations, due to difficulties in eliminating fish odor. New techniques obtained from the development of system biology have been applied to improve or manipulate the ruminal microbial community to modulate the bacterial population with bio-hydrogenation capacity [42]. Several studies have reported that the inclusion of linseed oil in cattle feed improves the FA profiles (decrease of *n*-6/*n*-3 ratio) and accumulates the ALA content by modulating the bio-hydrogenation capacity [43]. In our study, milking cows were fed with partially fermented cattle feed that contains two different sources of ALA (giant kelp and pomace of perilla seeds) resulting in a significant reduction in the *n*-6/*n*-3 FA ratio at the systemic levels, including meats, fats, and milk (data not shown). We cautiously speculate that our cattle feed may trigger the bypassing of bio-hydrogenation or the suppression of the bacterial population that have bio-hydrogenation capacity. Currently, it is under investigation whether our cattle feed induces a population shift of ruminal bacteria and suppresses the bio-hydrogenation capacity in rumens.

## 5. Conclusions

This study provides a novel insight that ALA-biofortified agricultural-products could be an alternative source of *n*-3 PUFA other than fish oil. The ALA-enriched butter can recapitulate the enhanced thermogenic energy expenditure, similarly to fish oil supplementation. The thermogenic activation of BAT was associated with the improved biosynthesis of *n*-3 LC PUFA, FA elongation/desaturation, and mitochondrial biogenesis. By thoroughly evaluating the effectiveness of ALA bio-fortified butter on thermogenesis in comparison with conventional butter and margarine, our study opens a new research avenue for designing health-promoting (or therapeutic) dairy products via manipulation of animal nutrition, metabolism, and presumably ruminal microbiome.

## Supplementary Materials

The following are available online at https://www.mdpi.com/2072-6643/12/1/136/s1, Figure S1. Supplementation with ALA-biofortified butter alters thermogenic genes expression in the BAT. Figure S2. Supplementation with ALA-biofortified butter increased fatty acid denaturation and elongation related genes expression in the BAT. Figure S3. Supplementation with ALA-biofortified butter did not alter cardiolipin (CL) synthase (*Crls1*) expression in the BAT. Table S1. Diet composition. Table S2. Primer sequences for qPCR. Table S3. List of primary antibodies.

## Abbreviations

ALA	*α*-linolenic acid
ARA	arachidonic acid
BAT	brown adipose tissue
Bu	conventional butter
DHA	docosahexaenoic acid
Elovl6	elongation of very long chain fatty acid-like family member 6
EPA	eicosapentaenoic acid
LA	linoleic acids
LC *n*-3 PUFA	long-chain omega 3 polyunsaturated fatty acids
Ma	margarine
n3Bu	*n*-3 PUFA enriched butter
SIRT3	Sirtuin 3
