# Supplementary material for: Alpha-Linolenic Acid-Enriched Butter Promotes Fatty Acid Remodeling and Thermogenic Activation in the Brown Adipose Tissue
Source: Nutrients. 2020 Jan 3;12(1):136. doi: 10.3390/nu12010136 (PMC7019653; doi:10.3390/nu12010136)
Supplement: Supplementary file 1 [file nutrients-12-00136-s001.pdf]

nutrients

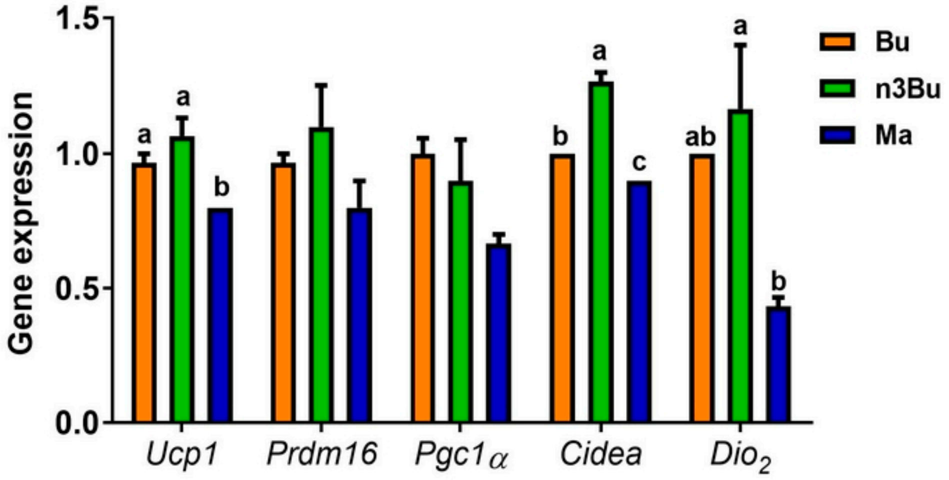

Figure S1. Supplementation with ALA-biofortified butter alters thermogenic genes expression in the BAT. mRNA expression levels of *Ucp1*, *Prdm16*, *Pgc1α*, *Cidea*, and *Dio2* from the pooled DNA (n=8 per group) with triplicated assay. All data represented as mean ± SEM. Treatments with different letters are significantly different from one another by one-way ANOVA (P<0.05).

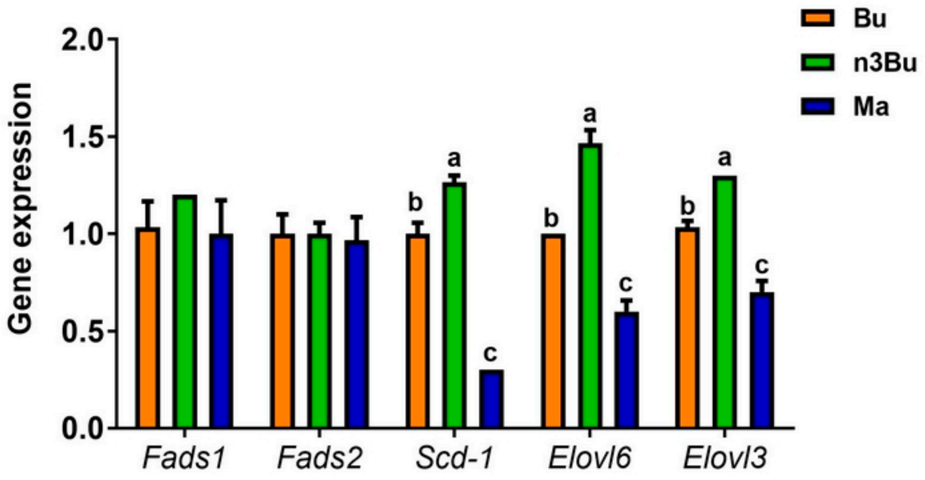

Figure S2. Supplementation with ALA-biofortified butter increased fatty acid denaturation and elongation related genes expression in the BAT. mRNA expression levels of *Fads1*, *Fads2*, *Scd-1*, *Elovl6*, and *Elovl3* from the pooled DNA (n=8 per group) with triplicated assay. All data represented as mean ± SEM. Treatments with different letters are significantly different from one another by one-way ANOVA (P<0.05).

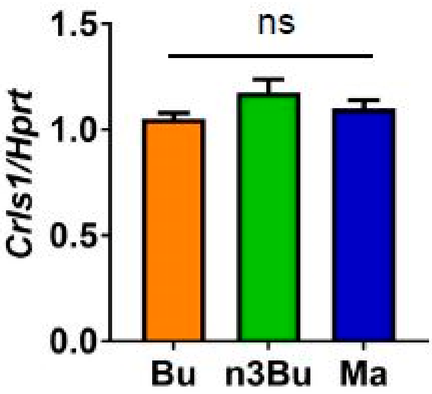

Figure S3. Supplementation with ALA-biofortified butter did not alter cardiolipin (CL) synthase (*Crls1*) expression in the BAT. mRNA expression *Crls1* in the BAT (n=4 of individual animals per group). All data represented as mean ± SEM. Treatments with different letters are significantly different from one another by one-way ANOVA (P<0.05).

Table S1. Diet composition of HF diet

**Ingredients (g)**	**Diet**		
	**Bu**	**n3Bu**	**Ma**
**Casein**	235	235	235
**L-cystine**	5	5	5
**Corn starch**	210	210	210
**Maltodextrin**	100	100	100
**Sucrose**	100	100	100
**Cholesterol**	2	2	2
**Cellulose**	50	50	50
**Mineral mix**	35	35	35
**Calcium carbonate**	30	30	30
**Vitamin mix**	10	10	10
**Choline bitartrate**	2.5	2.5	2.5
**Solid Fat**			
Hiland Butter	240	-	-
Sunseo Omega Butter		240	-
Land O Lake Margarine	-	-	240
**% calories from Fat**	45.4	45.4	45.4
**% calories from CHO**	34.4	34.4	34.4
**% calories from Protein**	20.2	20.2	20.2
**Kcal/g diet**	4.67	4.67	4.67

Table S2. Primer sequences for qPCR.

Primer	Primer sequence
Ucp1	F: 5′-AGGCTTCCAGTACCATTAGGT-3′<br>R: 5′-CTGAGTGAGGCAAAGCTGATTT-3′
Prdm16	F: 5′-CAG CAC GGT GAA GCC ATT C-3′<br>R: 5′-GCG TGC ATC CGC TTG TG-3′
Pgc1α	F: 5′-CCCTGCCATTGTTAAGACC-3′<br>R: 5′-TGCTGCTGTTCCTGTTTTC-3′
Cidea	F: 5′-TGCTCTTCTGTATCGCCCAGT-3′<br>R: 5′-GCCGTGTTAAGGAATCTGCTG-3′
Dio2	F: 5′-CAGTGTGGTGCACGTCTCCAATC-3′<br>R: 5′-TGAACCAAAGTTGACCACCAG-3′
Fads1	F: 5′-TCAGTCTTTGGCACCTCGAC-3′<br>R: 5′-TCCTTGCGGAAGCAGTTAGG-3′
Fads2	F: 5′-TCCTGTCCCACATCATCGTCATGG-3′<br>R: 5′-GCTTGGGCCTGAGAGGTAGCGA-3′
Scd-1	F: 5′-GGGACAGATATGGTGTGAAACTATG-3′<br>R: 5′-TTACAGACACTGCCCCTCAAC-3′
Elovl6	F: 5′-CGTAGCGACTCCGAAGATCAGCC-3′<br>R: 5′-AGCGTACAGCGCAGAAAACAGGA-3′
Elovl3	F: 5′-CTTCGAGACGTTTCAGGACTTAAG-3′<br>R: 5′-TCTGGCCAACAACGATGAG-3′
16S rRNA	F: 5′-CCGCAAGGGAAAGATGAAAGAC-3′<br>R: 5′-TCGTTTGGTTTCGGGGTTTC-3′
Hexokinase	F: 5′-GCCAGCCTCTCCTGATTTTAGTGT-3′<br>R: 5′-GGGAACACAAAAGACCTCTTCTGG-3′

Table S3. List of primary antibodies

Antibody	Host	Dilution	Company	Catalog no.
UCP1	Rabbit	1:1000	Abcam	Ab155117
PRDM16	Mouse	1:1000	Santa Cruz	Sc130243
CD11c	Rabbit	1:1000	Cell Signaling	97585
F4/80	Rabbit	1:1000	Abcam	Ab6640
SCD1	Rabbit	1:1000	Cell Signaling	2794
Elovl6	Rabbit	1:1000	Abcam	Ab69857
VDAC1	Rabbit	1:1000	Cell Signaling	4661
PDH	Rabbit	1:1000	Cell Signaling	3205
OxPhos	Mouse	1:250	Abcam	ab110413
SIRT3	Rabbit	1:1000	Cell Signaling	5490
β-actin	Rabbit	1:1000	Cell Signaling	4967
